# Left Ventricular Rupture as a Complication of Anterior Wall Myocardial Infarction on Computed Tomography Angiography and Ventriculography

**DOI:** 10.3390/diagnostics11030525

**Published:** 2021-03-16

**Authors:** Paweł Gać, Piotr Macek, Andrzej Szczepański, Rafał Poręba

**Affiliations:** 1 Centre for Diagnostic Imaging, 4th Military Hospital, Weigla 5, PL 50-981 Wroclaw, Poland; 2 Department of Hygiene, Wroclaw Medical University, Mikulicza-Radeckiego 7, PL 50-368 Wroclaw, Poland; 3 Department of Internal and Occupational Diseases, Hypertension and Clinical Oncology, Wroclaw Medical University, Borowska 213, PL 50-556 Wroclaw, Poland; piotr.macek@umed.wroc.pl (P.M.); rafal.poreba@umed.wroc.pl (R.P.); 4 Centre for Heart Diseases, 4th Military Hospital, Weigla 5, PL 50-981 Wroclaw, Poland; szczepanskiwroc@gmail.com

**Keywords:** left ventricular rupture, myocardial infarction, computed tomography angiography

## Abstract

Rupture of the free wall of the left ventricle, rupture of the interventricular septum and acute mitral regurgitation are mechanical complications of myocardial infarction. They are rare; left ventricular rupture occurs in about 2–4% of patients with myocardial infarction. We present the case of an 85-year-old woman with an anterior wall infarction complicated by left ventricular rupture. We present diagnostic images of pathology visualized by computed tomography angiography, performed in order to exclude aortic dissection as the cause of the presence of fluid in the pericardial sac. Images from ventriculography are also presented. Summing up, during the diagnostic and therapeutic process of acute coronary syndrome, it is important to bear in mind the risk of possible complications, such as left ventricular rupture.

Ischemic heart disease is one of the leading causes of death in the general population, especially in developed countries and in the elderly population. Much progress has been made in the treatment of acute coronary syndromes in recent years. However, complications of myocardial infarction remain a diagnostic and therapeutic challenge [1].

We present the case of an 85-year-old woman with left ventricular rupture as a complication of anterior wall myocardial infarction. We present diagnostic images of pathology, visualized by computed tomography angiography performed to exclude aortic dissection as the cause of the presence of fluid in the pericardial sac. Images from ventriculography are also presented.

An 85-year-old female patient was brought by a medical emergency team to the hospital emergency department (ED) with a diagnosis of myocardial infarction. In her medical history, the patient reported weakness for 2 days, and on the day of calling for an ambulance she reported an episode of collapse. ECG showed a picture of ST-segment elevation myocardial infarction in the area of the anterior and inferior wall of the heart. Laboratory tests revealed increased high-sensitivity troponin I (1.78 ng/L), NTproBNP (8752 pg/mL) and CRP (28.3 mg/L). Bedside echocardiography in an ED setting revealed pericardial effusion, a layer about 2.0 cm thick along the right ventricular free wall in the subcostal view and up to 3.0 cm along the right ventricular free wall in the apical view, partially intensely saturated, with collapse of the right ventricular and right atrial free wall—an image suggesting cardiac tamponade. Moreover, akinetic cardiac apex and left ventricular systolic dysfunction with an ejection fraction of 35% were observed. The ascending aorta was visualized only in the root area, with no conclusive signs of dissection. Since it was not possible to exclude the ascending aortic section, and cardiac tamponade was suspected, a decision was made to supplement diagnostics with computed tomography angiography (CTA) of the thoracic aorta.

ECG-gated CT angiography of the thoracic aorta revealed a trileaflet aortic valve, a normal-sized aortic root (diameter about 3.2 cm), numerous atherosclerotic plaques in the coronary arteries, with maintained ostium patency, and normal-sized thoracic aorta with numerous atherosclerotic plaques of various morphotic types (with a diameter in the ascending section, aortic arch and descending section of 3.0 cm, 2.5 cm and 2.2 cm, respectively, Figure 1A).

No signs of dissection were revealed over the entire length of the thoracic aorta. CTA revealed a presence of bloody fluid in the pericardial sac, and a maximum layer thickness of about 2.0–2.5 cm along the diaphragmatic surface of the heart (Figure 1B). The examination also revealed the presence of myocardial rupture in the area of the apical inferior segments of the left ventricular wall, with a visible active leakage of contrasted blood into the pericardial sac (Figure 1C,D).

The patient underwent coronary angiography, showing diffuse spasm of coronary vessels, probably because of the used catecholamines (Figure 2A). 

After the administration of nitrate i.c., tight stenosis in the seventh segment of the left anterior descending artery was revealed (Figure 2B). Narrowing up to 70% was found in other vessels. Vessel flow was maintained in both the left and the right coronary artery. Ventriculography confirmed a mechanical complication of the myocardial infarction-pseudoaneurysm in the left ventricular apical segment (Figure 2C,D).

A decision was made to perform urgent cardiac surgery, to which the patient gave her informed refusal, despite receiving conclusive information about the bad prognosis in the event of refusal to undergo surgical treatment. The patient died of PEA cardiopulmonary arrest.

There are three main known mechanical complications of myocardial infarction: left ventricular free wall rupture, ventricular septal rupture and acute mitral regurgitation. Mechanical complications of myocardial infarction are rare. Left ventricular free wall rupture occurs in 2 to 4% patients with myocardial infarction. It is characterized by a high mortality rate, with the most common mechanical complication of myocardial infarction resulting in sudden cardiac death [2].

Left ventricular wall rupture occurs mainly in patients aged over 65, and more frequently among women. In most cases, it affects patients with arterial hypertension. Typically, it occurs during the first week, usually on the fourth or fifth day, after myocardial infarction. It is more common during the first transmural myocardial infarction [3].

Left ventricular free wall rupture may be of two kinds. Complete rupture causes cardiac tamponade and results in sudden cardiac death. The rupture may also involve the formation of ventricular pseudoaneurysm when the blood outflow to the pericardial sac is inhibited by the formation of thrombus and the sealing function of the pericardium [4].

The most important diagnostic method for left ventricular free wall rupture is transthoracic echocardiography; the presence of reduced myocardial wall thickness, hemopericardium or epicardial clots, and cardiac tamponade, are the most relevant findings. Diagnosis of left ventricular pseudoaneurysm can be made by several imaging techniques, including echocardiography, computed tomography, ventriculography and magnetic resonance imaging (MRI). Echocardiography is the first-line imaging modality in suspected left ventricular pseudoaneurysm. However, contrast ventriculography has a good likelihood of establishing a definitive diagnosis [5].

Only in the publications of Onoda et al. and Redfern et al. computed tomography images documenting left ventricular free wall rupture were shown [6,7]. The CTA images in our study were obtained via a protocol with gated acquisition by ECG during the diastolic phase, in thin slices of 0.6 mm, with good contrast of the left heart cavities and aorta (>300 HU) and moderate right heart cavity contrast (100–200 HU).

## Figures and Tables

**Figure 1:**
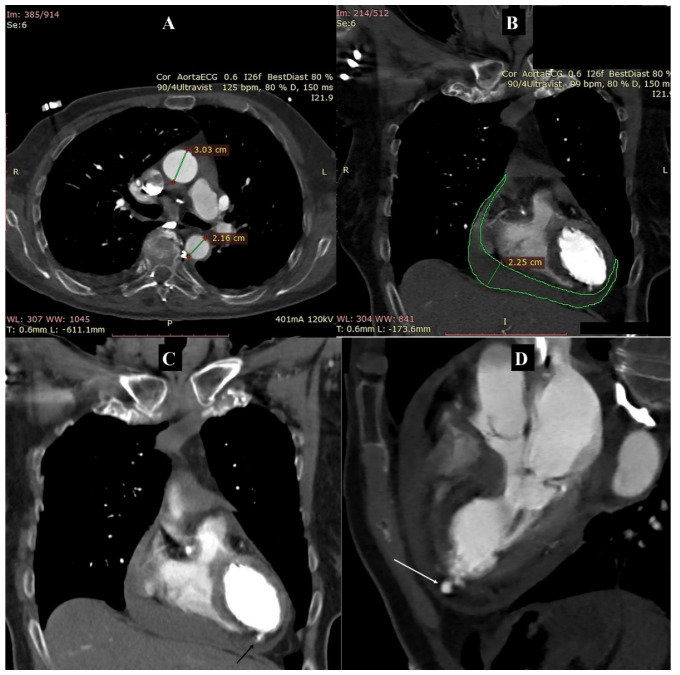
Computed tomography angiography of the thoracic aorta: (**A**) Axial reconstruction. Normal-sized thoracic aorta. Measurements: diameter of the ascending and descending aorta. (**B**) MPR reconstruction. Coronal view. Fluid in the pericardial sac. Contour: lamina of the pericardium. Measurement: maximum thickness of the fluid layer. (**C**) MIP reconstruction. Coronal view. Myocardial rupture with a visible active leakage of contrasted blood into the pericardial sac (arrow). (**D**) MPR reconstruction. Three-chamber view. Myocardial rupture with a visible active leakage of contrasted blood into the pericardial sac (arrow).

**Figure 2:**
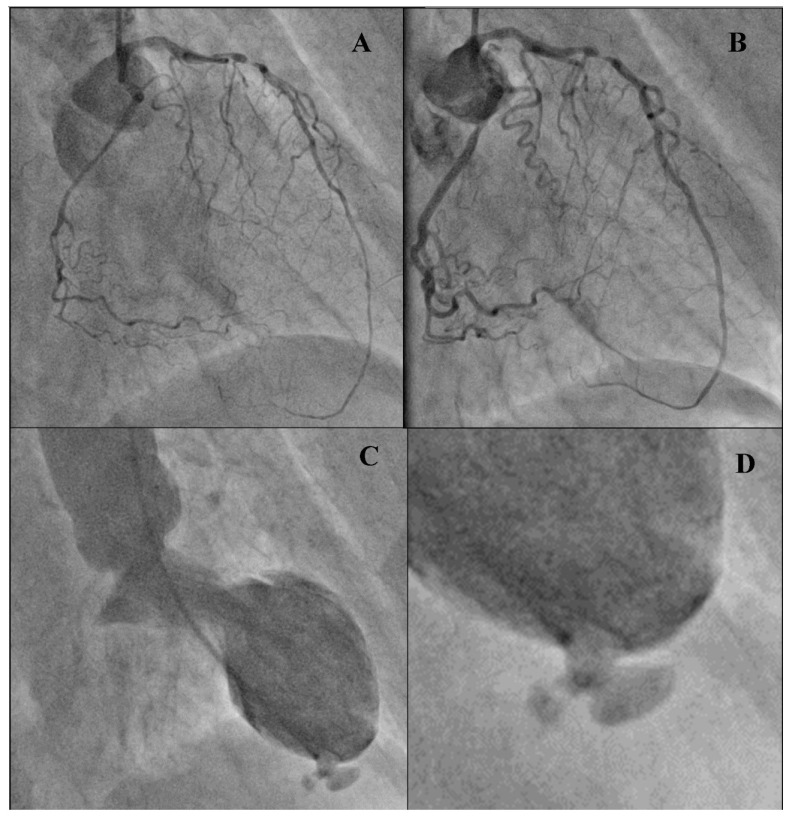
Coronary angiography and ventriculography: (**A**) Coronary angiogram of left artery-diffuse spasm of coronary vessels (30 deg. RAO). (**B**) Coronary angiogram of left artery after administration 200 μg NTG (nitroglycerin) i.c. (intracoronary)–critical stenosis in seventh segment of the left anterior descending artery (LAD). (**C**) Ventriculography (30 deg. RAO), pig tail catheter inside left ventriculum. Systolic phase: akinesis of antero-lateral, apical and diaphragmatic segments. Pseudoaneurysm outgrowing from posterior, apical segment. (**D**). Enlargement picture with pseudoaneurysm.
